# Supplementary material for: The Impact of Yeast Encapsulation in Wort Fermentation and Beer Flavor Profile
Source: Polymers (Basel). 2023 Mar 31;15(7):1742. doi: 10.3390/polym15071742 (PMC10096922; doi:10.3390/polym15071742)
Supplement: Supplementary file 1 [file polymers-15-01742-s001.zip › polymers-2261204-supplementary.pdf]

polymers

Supplementary material

Article

The Impact of Yeast Encapsulation in Wort Fermentation and Beer Flavor Profile

Angie D. Bolanos-Barbosa [1], Cristian F. Rodríguez [2], Olga L. Acuña [1], Juan C. Cruz [2,*] and Luis H. Reyes [1,*]

[1] Product and Process Design Group (GDPP), Department of Chemical and Food Engineering, Universidad de Los Andes, Bogotá 111711, Colombia
[2] Department of Biomedical Engineering, Universidad de Los Andes, Bogotá 111711, Colombia
* Correspondence: jc.cruz@uniandes.edu.co (J.C.C.); lh.reyes@uniandes.edu.co (L.H.R.); Tel.: +57-1-339-4949 (ext. 1789) (J.C.C.); +57-1-339-4949 (ext. 1702) (L.H.R.)

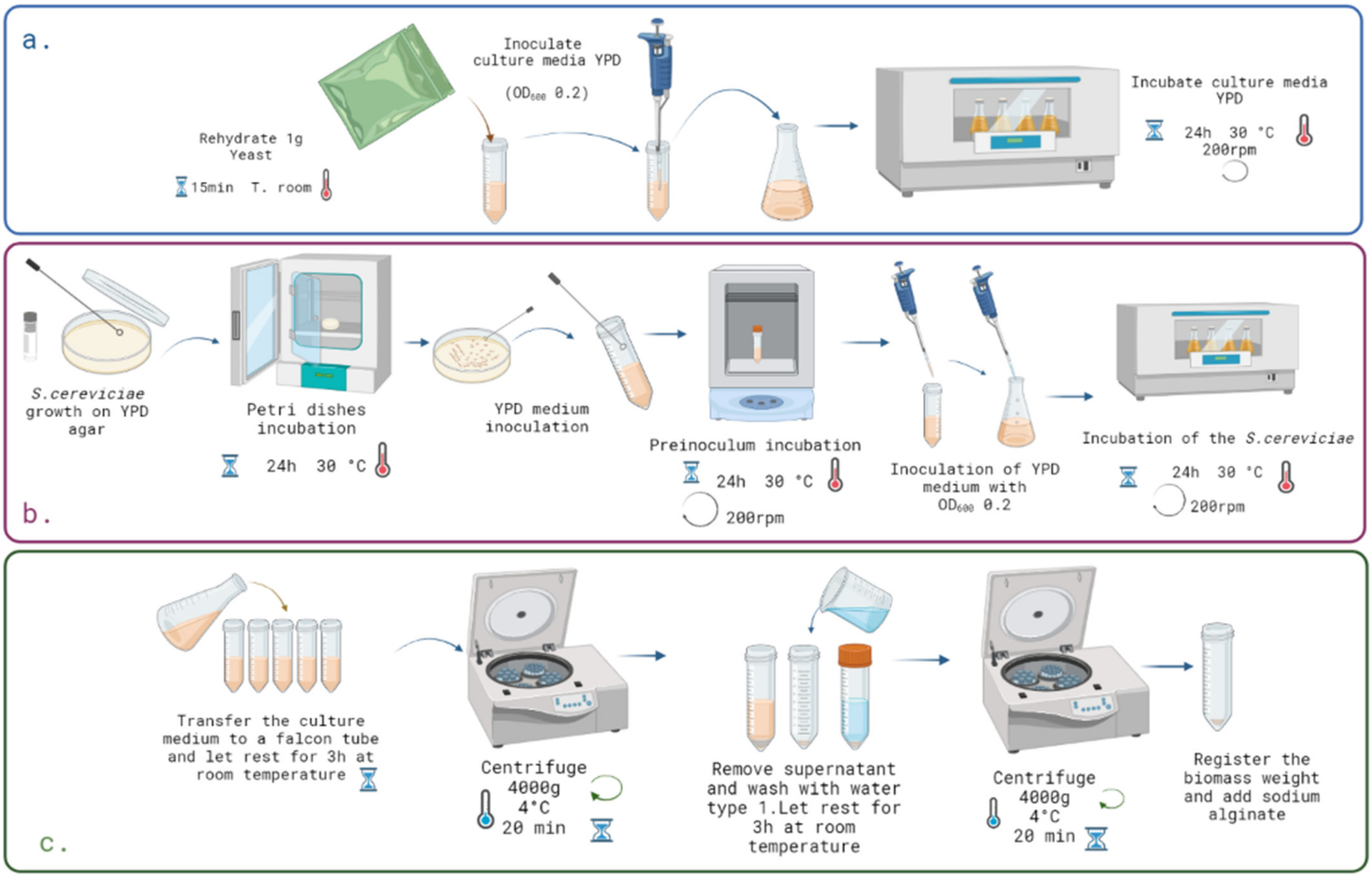

Figure S1. Process for obtaining biomass for encapsulation: **(a)** Growth process for the BE-134, K-97, and US-05 commercial strains, **(b)** Growth process for the CW-12 wild strain, **(c)** After the growth processes for all the strains, the culture was transferred to Falcon tubes to centrifuge and rinse them. The supernatant was discarded, while the biomass was collected for further use (Created in BioRender.com).

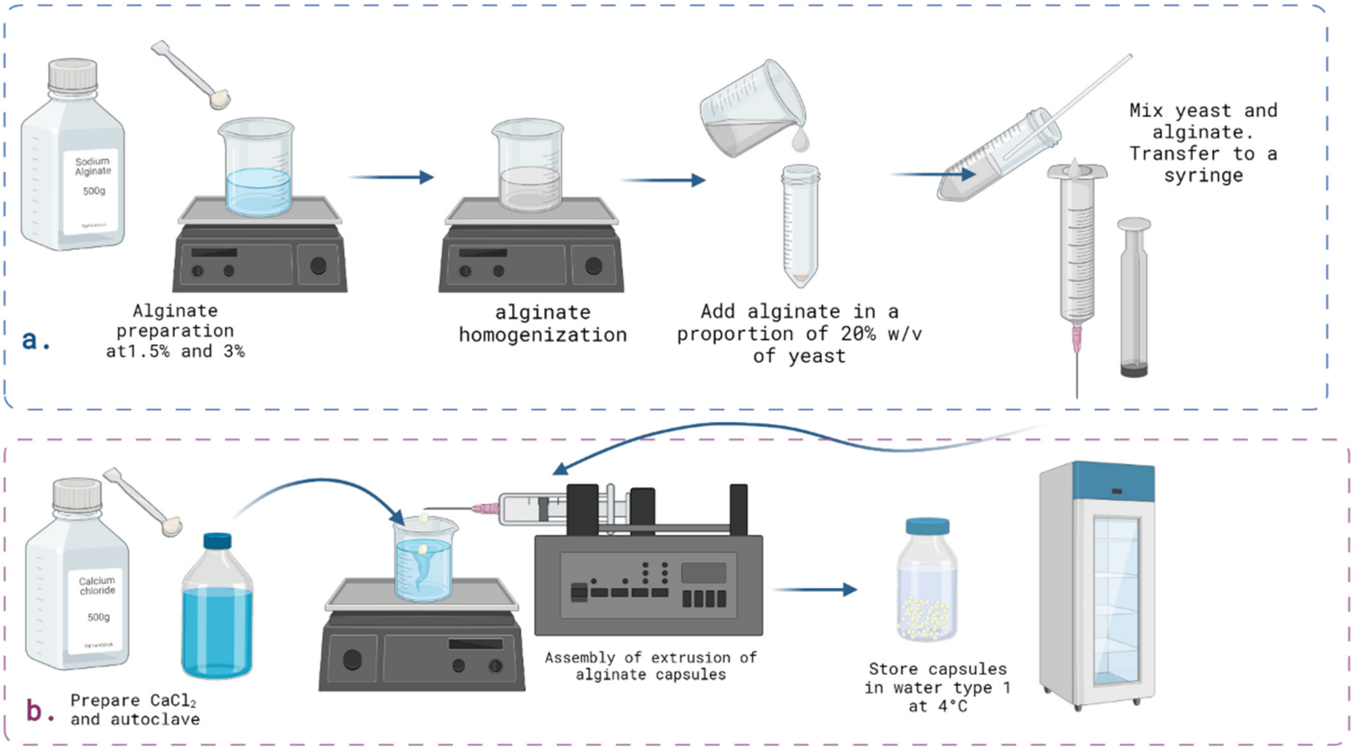

Figure S2. Encapsulation process for yeast in calcium alginate, **(a)** Yeast-sodium alginate mixture preparation, **(b)** Assembly for producing calcium alginate capsules containing yeast.

Diffusion simulation

For this early approach to establishing the diffusivity, an experimental test was performed in which 50 mM methylene blue was encapsulated using each of the 6 formulations proposed for this study. Ten capsules were added to 5 mL of Type 1 water in a 15-mL Falcon tube, and the concentration of methylene blue in the medium was monitored using spectrophotometry over a 6-hour period. The experimental data obtained was used to fit a 2D mathematical model simulated with the COMSOL Multiphysics 6.0® software (COMSOL Inc., Stockholm, Sweden).

The release was studied with a mass transport approach governed by Fick's law. The transport of solute was assumed in a porous medium governed by Equation (S1).

$$\frac{\partial(\varepsilon_p c)}{\partial t} + \nabla J_i = 0, \quad \text{(S1)}$$

Copyright: © 2023 by the authors. Licensee MDPI, Basel, Switzerland. This article is an open access article distributed under the terms and conditions of the Creative Commons Attribution (CC BY) license (https://creativecommons.org/licenses/by/4.0/).

Where ε_p is the porosity, c is the concentration, and J is the diffusive flux vector governed by Equation (S2).

$$J = -(D_e)\nabla C_i, \quad \text{(S2)}$$

Where D_e is the effective diffusion, which was determined by multiplying the free flow diffusion coefficient by the tortuosity in Equation (S3).

$$D_e = -\frac{\varepsilon}{\tau} D_f, \quad \text{(S3)}$$

The tortuosity was related to the porosity, employing the Millington-Quirk correlations in Equation (S4).

$$\tau = \varepsilon^{-\frac{1}{3}}, \quad \text{(S4)}$$

The model's equations were solved by simulations conducted via a time-dependent study with a PARDISO solver. Figure S3 shows the computational domain for configuration 1 meshed with 260466 domain elements and 4148 boundary elements. These meshing levels allowed convergence (Data not show). The boundary conditions imposed to solve the equation were the axial symmetry, the no-flow boundary, and the properties of the capsules. The axial symmetry, and the no flux, represent a no mass flux across the boundary. The boundary conditions and the variables considered for these simulations are shown in Figure S3. The model analyzed the effective diffusion of the capsules with different concentrations. The parameters of the simulation are porosity and radius of each capsule.

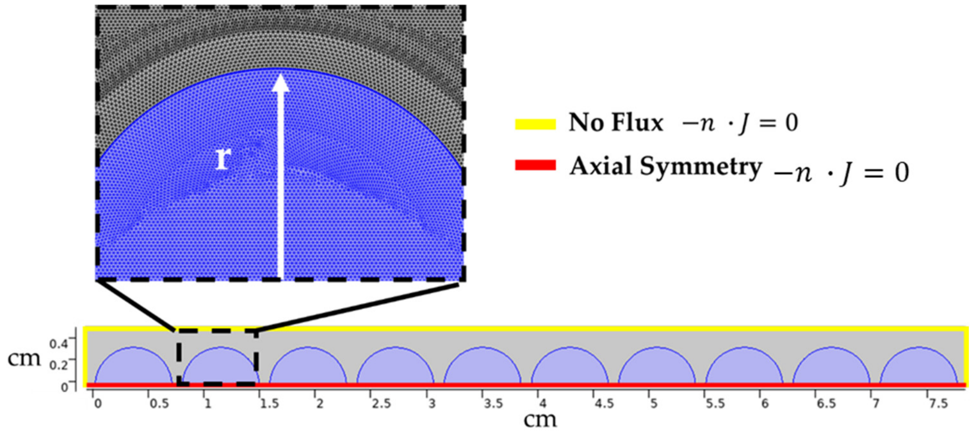

Figure S3. Boundary conditions, variables, and mesh for the simulations. Where axial symmetry is shown in red, no flow in yellow, and r is the radius of the capsules.

The model analyzed the effective diffusion of the capsules with different concentrations. All the parameters of the simulation are in Table S1.

Table S1. Parameters for the diffusion simulations.

Concentrations	Parameter	Value	Units
1.5% Alginate and 0.2 M calcium chloride	Porosity (ε)	0.574	Dimensionless
	Radius (r)	1.501	mm
1.5% Alginate and 0.1 M calcium chloride	Porosity (ε)	0.588	Dimensionless
	Radius (r)	1.7	mm
1.5% Alginate and 0.05 M calcium chloride	Porosity (ε)	0.574	Dimensionless
	Radius (r)	1.75	mm
3% Alginate and 0.2 M calcium chloride	Porosity (ε)	0.552	Dimensionless
	Radius (r)	1.7	mm
3% Alginate and 0.1 M calcium chloride	Porosity (ε)	0.661	Dimensionless
	Radius (r)	1.75	mm
3% Alginate and 0.05 M calcium chloride	Porosity (ε)	0.654	Dimensionless
	Radius (r)	1.75	mm

Figure S4 shows the results of the experimental diffusion tests. In these tests, the capsules were generated at the different formulations of the experimental design shown in Figure S7, with an initial concentration of 50 mM methylene blue. The size of the capsules

in the multiphysics model was considered to adjust the simulations and identify how the effective diffusion coefficient changed at different alginate concentrations. Figure S5a shows the results of how the effective diffusion coefficients of the capsules vary according to how the alginate concentrations vary, and Figure S5b shows how the effective diffusion coefficients of the capsules vary according to how the concentrations of calcium chloride change. These coefficients were determined by adjusting the proposed multiphysics model, with the results obtained experimentally shown in Figure S4.

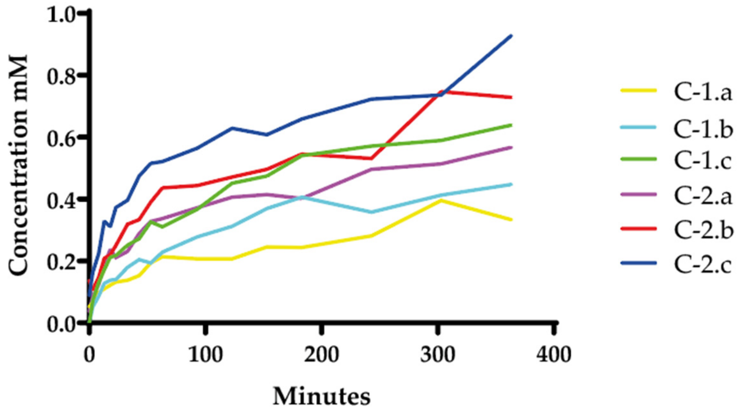

Figure S4. Experimental diffusion study. The concentration of methylene blue recorded in a study of 363 minutes is presented.

The diffusivity coefficient of methylene blue in water is approximately 4.6 × 10^{-10} [56], while the simulation results indicate a value of 1.29 × 10^{-11}, which allows one to infer that the alginate capsule diminished the diffusivity value by 97% for this substance. Additionally, as illustrated in Figure S5, it was observed that the diffusivity increases with an increase in the concentration of calcium chloride. This can be attributed to the higher availability of calcium ions. Also, a small increase of 0.19% in this parameter was observed when going from 1.5 to 3% alginate concentrations, which can be considered insignificant and goes against the expected behavior that indicates that diffusivity must decrease as the alginate concentration increases [57]. However, it's necessary to evaluate this model in its application to the sugars present in the fermentation medium.

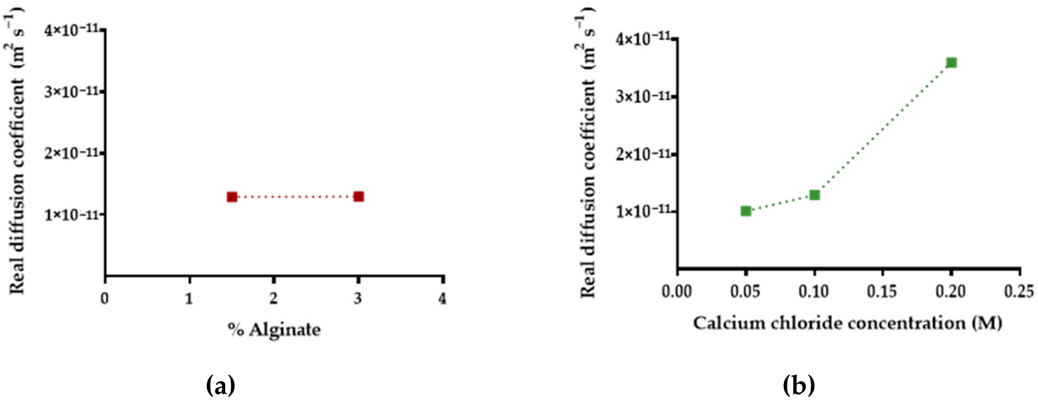

Figure S5. Effective diffusion coefficients of the capsules. **(a)** Variation in the effective diffusion coefficient by varying the alginate concentration. **(b)** Variation in the effective diffusion coefficient by varying the calcium chloride concentration.

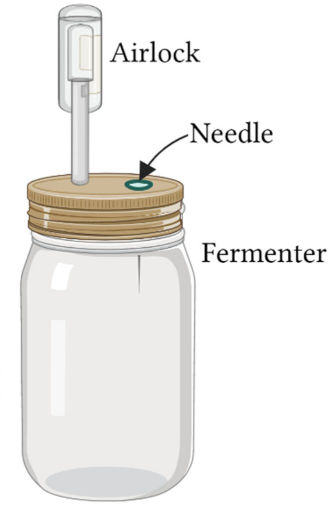

Figure S6. Fermenter assembly representation.

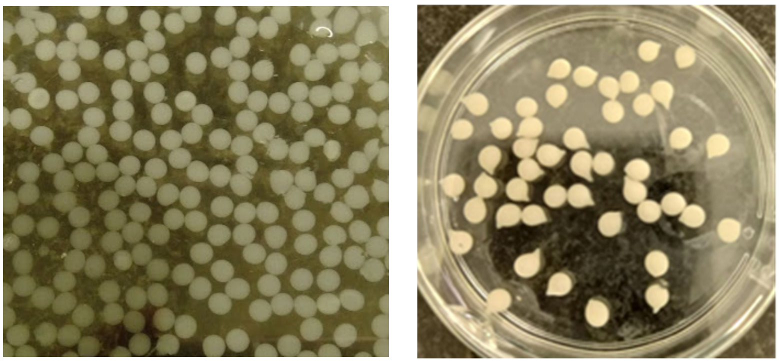

(**a**) (**b**)

Figure S7. Shape comparison (**a**) 1.5% alginate capsules (**b**) 3% alginate capsules.

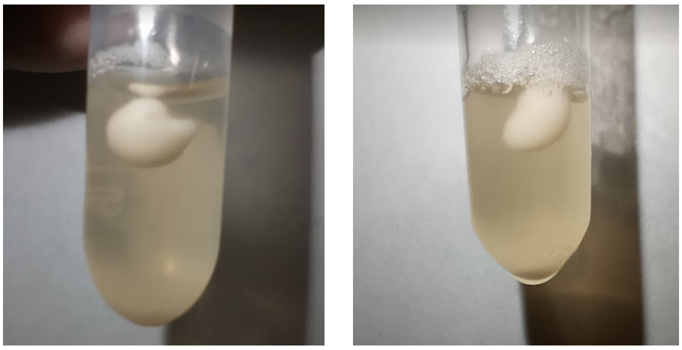

(**a**) (**b**)

Figure S8. Macroscopic surface alterations in 1.5% alginate capsules during the swelling test. **(a)** Marked protuberance on the surface of the capsule, **(b)** Elongation and decrease of sphericity of the capsule.

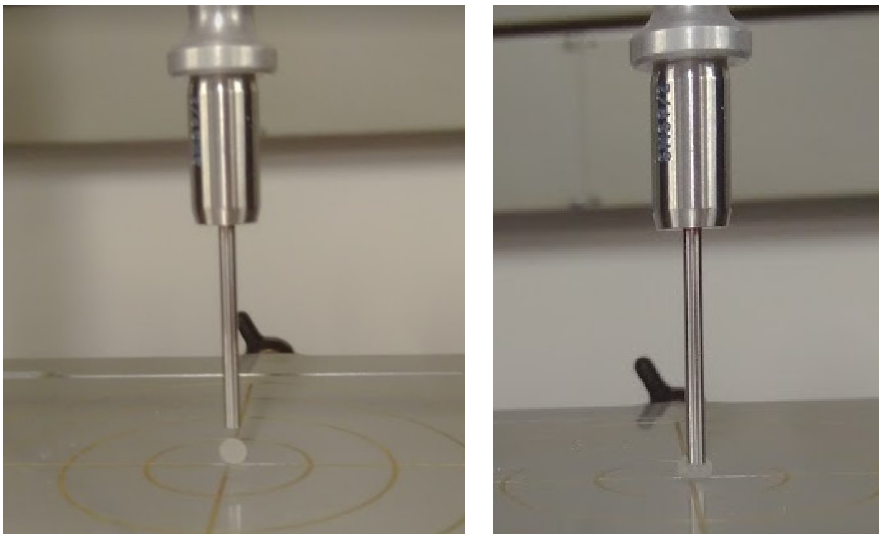

(**a**) (**b**)

Figure S9. Breaking force and elasticity test. **(a)** Assembly ready to test **(b)** test under execution.

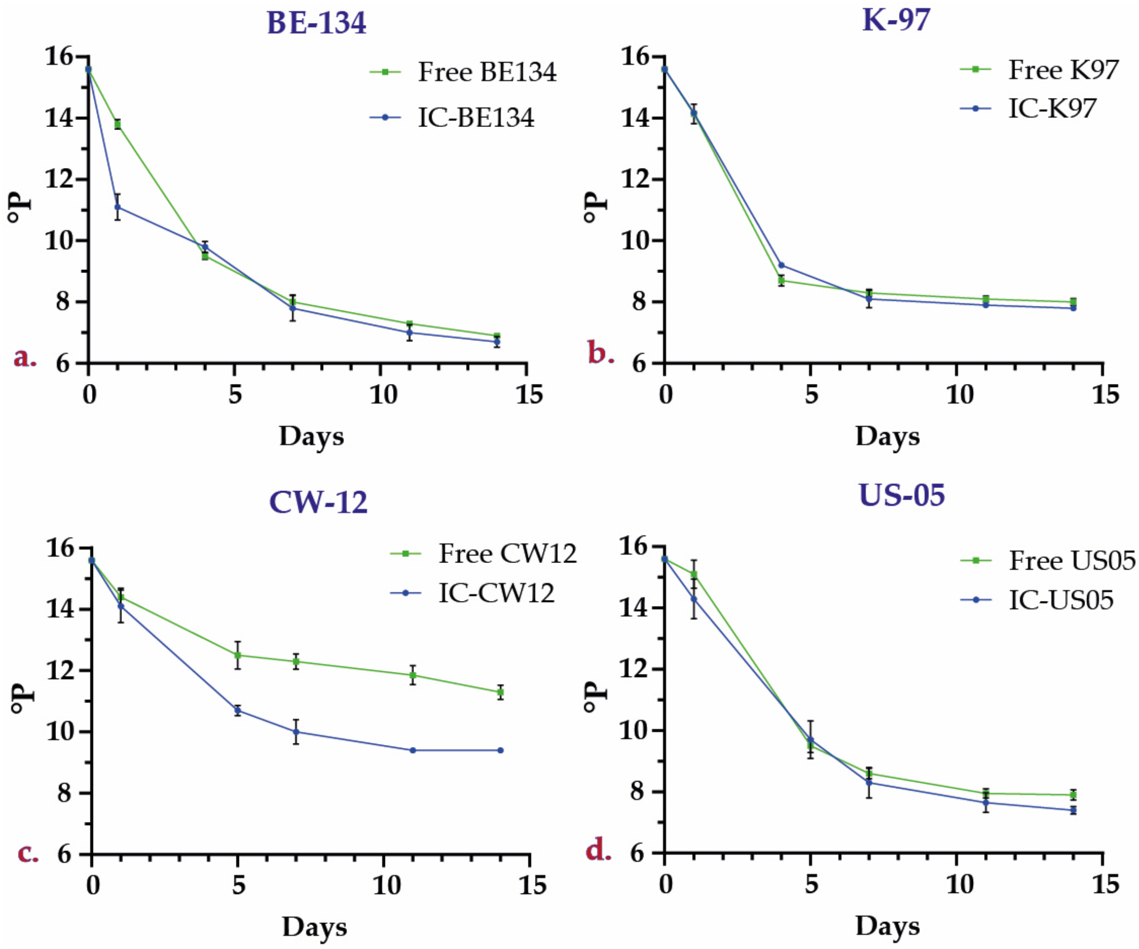

Figure S10. Comparative °P monitoring during fermentation for encapsulated and free yeast cells for **(a)** *S. cerevisiae* BE-134 yeast, **(b)** *S. cerevisiae* K-97 yeast, **(c)** *S. cerevisiae* CW-12 yeast, and **(d)** *S. cerevisiae* US-05 yeast.

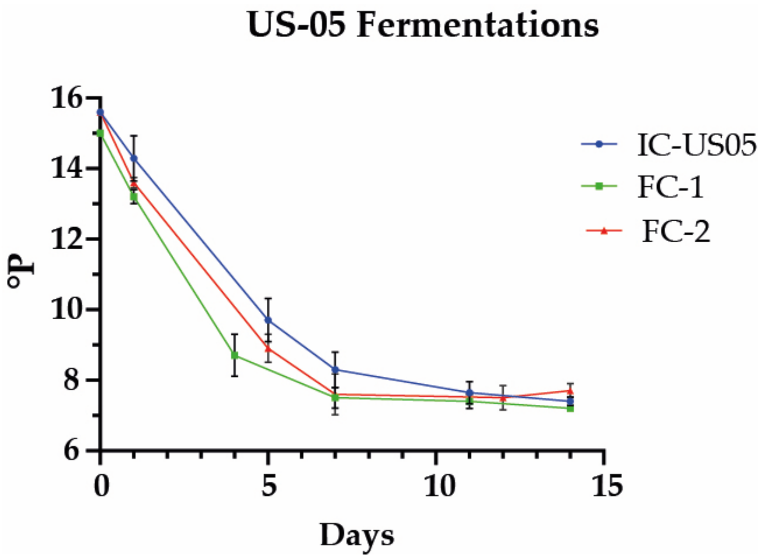

Figure S11. °P monitoring of fermentation with reused encapsulates after each repeated batch.
